# Efficient Hydrogen Production via Photodehydrogenation of Ammonia Borane Using Embedded CdO/ZnO Nanoparticles in Polyurethane Nanofibers

**DOI:** 10.3390/polym17040443

**Published:** 2025-02-08

**Authors:** Isam Y. Qudsieh, Ibrahim M. Maafa, Ayman Yousef, Ahmed Abutaleb, Saleh M. Matar, M. M. El-Halwany

**Affiliations:** 1 Department of Chemical Engineering, College of Engineering and Computer Sciences, Jazan University, Jazan 45142, Saudi Arabia; isamyq@jazanu.edu.sa (I.Y.Q.); azabutaleb@jazanu.edu.sa (A.A.); sel-saidmatar@jazanu.edu.sa (S.M.M.); 2 Engineering and Technology Research Center, Jazan University, P.O. Box 114, Jazan 82817, Saudi Arabia; 3 Department of Mathematics and Physics Engineering, Faculty of Engineering, Mansoura University, El-Mansoura 35516, Egypt; mmhalwany@mans.edu.eg; 4 Department of Chemical Engineering, Faculty of Engineering, Mansoura National University, El-Mansoura 35516, Egypt

**Keywords:** electrospinning, Cd/ZnO, polymer, photohydrolysis, ammonia borane, hydrogen production

## Abstract

The urgent global demand for sustainable green energy solutions has recognized hydrogen (H_2_) as a viable green energy carrier. This study explores the efficient production of H_2_ as a potential source of sustainable, environmentally friendly, high-energy-density fuel characterized by eco-friendly burning by-products. The research focuses on the photohydrolysis reaction of ammonia borane (AB), utilizing CdO-doped ZnO nanoparticles (NPs) embedded in polyurethane (PU) nanofibers (CdO/ZnO NPs@PU NFs) as a novel photocatalyst. Three different amounts of CdO/ZnO NPs were loaded onto PU NFs. The synthesized CdO/ZnO NPs@PU NFs exhibited good photocatalytic performance under visible light, producing approximately 67 mL of H_2_ from 1 mmol of AB in 15 min with the sample containing the highest loading of CdO/ZnO NPs@PU NFs. This impressive photocatalytic performance is attributed to the synergistic effects of CdO and ZnO, which enhance charge carrier separation and broaden bandgap absorption in the visible spectrum. Kinetic studies demonstrated that the reaction exhibited first-order kinetics regarding catalyst dosing and zero-order kinetics concerning AB concentration, with an activation energy (E_a_) of 32.28 kJ/mol. The results position CdO/ZnO NPs@PU NFs as effective photocatalysts for H_2_ photogeneration under visible light irradiation.

## 1. Introduction

Industrial expansion and a growing population are driving the increasing global energy demand, necessitating a crucial shift toward sustainable energy sources [1]. Fossil fuels, which have been the predominant energy source over the last century, have significantly contributed to greenhouse gas emissions and environmental degradation [2]. Consequently, renewable energy sources such as solar, wind, and hydrogen (H_2_) are regarded as the most feasible alternatives for reducing our reliance on fossil fuels and achieving long-term sustainability [3,4]. Among these alternatives, H_2_ has garnered considerable interest due to its clean combustion, yielding only water as a by-product. Furthermore, H_2_ possesses a substantial energy density of approximately 120 MJ/kg, nearly three times that of gasoline (44 MJ/kg), making it an effective energy carrier for diverse applications, including fuel cells and industrial processes [5]. Nevertheless, despite its potential, H_2_ faces numerous problems concerning production, storage, and transportation [4]. Conventional H_2_ generation processes, such as steam methane reforming, are energy-intensive and produce carbon dioxide as a by-product [6]. Therefore, it is imperative to develop environmentally sustainable and energy-efficient methods for H_2_ production. Additionally, the low volumetric energy density of H_2_ presents significant challenges in achieving compact and effective storage.

Solid H_2_ storage compounds, such as ammonia borane (AB, NH_3_BH_3_), are considered promising candidates for H_2_ storage applications. AB is a viable chemical H_2_ storage medium due to its substantial H_2_ content (19.6 wt%), low molecular weight (MW) (30.87 g/mol), and advantageous stability at ambient temperatures [7]. It can generate H_2_ via hydrolysis in the presence of H_2_O, rendering it a highly attractive choice for H_2_ storage and release. The dehydrogenation of AB can yield up to three equivalents of H_2_ per mole of AB, as illustrated in the following reaction (1):

(1) $${NH}_{3}{BH}_{3}+3H_{2}O\;\overset{\mathrm{Catalyst}}{\to }\;{NH}_{3}+H_{3}{BO}_{3}+3H_{2}↑$$

Appropriate catalytic materials are required for enhancing the H_2_ evolution rate and overall performance. The high cost of precious metals has prompted increasing attention to the development of alternative catalytic materials that are abundant, economical, and effective. In recent years, numerous catalytic transition metals and their oxides have been investigated for the dehydrogenation of AB [8,9].

Since the first report on the use of photocatalytic materials in the photohydrolysis of AB [10] by Ayman et al. and his research group in 2012, the scientific community has explored various aspects of photocatalysis, resulting in numerous subsequent publications. Simagina et al. [11] demonstrated that adding colloidal Ag to TiO_2_ significantly enhanced the photocatalytic process for H_2_ production from AB. Specifically, the incorporation of 0.06% Ag into TiO_2_ improved the separation of photogenerated electron–hole pairs, thereby reducing recombination and enhancing photocatalytic activity under UV light. The Ag nanoparticles (NPs) improved electron transfer efficiency, resulting in 71% H_2_ production from the photodehydrogenation of AB. Jin Song et al. [12] studied the photocatalytic production of H_2_ from AB utilizing Co NPs supported on a CeVO_4_/CeO_2_ heterostructure under visible light irradiation. The prepared co-catalyst (CeVO_4_/CeO_2_) in a 2:1 molar ratio demonstrated a turnover frequency (TOF) of 90.9 min^−1^ at 25 °C. This improved photocatalytic performance is attributed to the effective separation of electron–hole pairs and an increased electron density on the Co NPs. Manyi Gao et al. [13] synthesized monodisperse Ni NPs of varying sizes (3.2, 5.4, and 7.2 nm) via a facile liquid-phase self-assembly method and embedded them onto g-C_3_N_4_ nanosheets. The resulting composite demonstrated outstanding photocatalytic performance for the photohydrolysis of AB under visible light, with the optimal Ni nanoparticle size of 3.2 nm achieving a TOF of 18.7 mol (H_2_) mol (catalyst)^−1^ min^−1^ and an activation energy (Ea) of 36 kJ mol^−1^.

Zinc oxide (ZnO) has been extensively investigated as a photocatalyst due to its semiconducting characteristics, chemical stability, and environmental compatibility [14,15]. Nonetheless, its large band gap (3.37 eV) limits its efficacy under visible light exposure [16,17]. Doping ZnO with cadmium oxide (CdO), which has a smaller band gap of 2.3 eV, has been shown to enhance visible light absorption and charge separation, thereby improving overall photocatalytic efficacy [18,19,20]. Despite these developments, issues such as nanoparticle aggregation and limited stability continue to undermine the efficacy of catalytic systems [20,21]. The agglomeration of catalytic NPs negatively impacts the catalytic performance in H_2_ production from AB [22]. To address this issue, it is crucial to immobilize the NPs on support matrices such as metal oxides, nanocarbon, polymers, or metal-organic frameworks [23]. These supports not only provide a stable environment for the NPs, preventing their agglomeration, but also enhance their dispersion and accessibility to reactants. Utilizing appropriate support materials can improve the catalytic activity and durability of the NPs, resulting in more efficient H_2_ production processes. Our research group has employed carbon nanofibers and polymer membranes for catalytic H_2_ synthesis from AB and sodium borohydride. Polymer-supported catalysts facilitate separation and recycling, which are essential for the long-term sustainability of H_2_ production systems. Electrospinning for the production of polymer-supported nanofibers has demonstrated a substantial enhancement in photocatalytic efficiency, attributed to increased surface area and improved dispersion of the active catalyst. To further enhance the catalytic activity and stability of the CdO-ZnO photocatalyst, it was immobilized on polyurethane (PU) NFs.

This work presents a distinctive approach to overcoming existing constraints by immobilizing CdO/ZnO NPs onto electrospun PU nanofibers. This approach reduces NP agglomeration, promotes the dispersion of active sites, and accelerates charge transfer by forming heterojunctions. The CdO/ZnO NPs@PU NFs composite was assessed for H_2_ generation from AB under visible light exposure. The synthetic photocatalyst exhibited remarkable H_2_ generation efficiency, achieving stoichiometric H_2_ release with outstanding stability and reusability over several cycles. This research advances photocatalytic H_2_ generation by offering a cost-effective, stable, and highly efficient solution.

## 2. Experimental

### 2.1. Photocatalyst Preparation

**Scheme 1:**
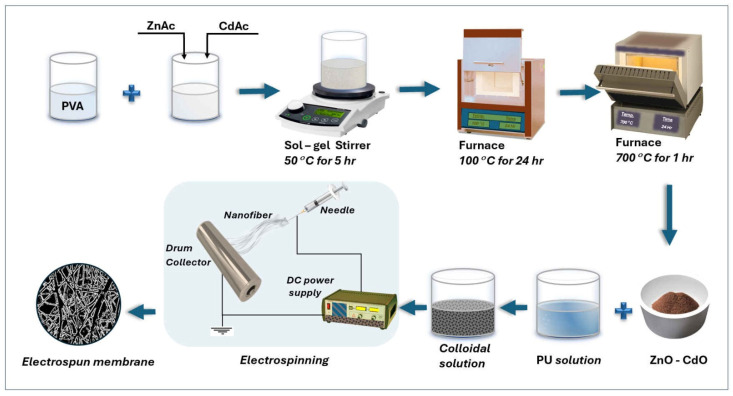
Diagram of CdO-doped ZnO NPs@PU NFs preparation.

In a typical procedure, cadmium acetate dihydrate (CdAc, 99.0% purity) and zinc acetate dihydrate (ZnAc, 99.0% purity) were dissolved in 5 mL of distilled water in a 0.5:1 molar ratio. The resulting mixture was then combined with 15 g of an aqueous poly (vinyl alcohol) (PVA, MW of 65,000 g/mol) solution (10 wt%). The solution was stirred for 5 h at 50 °C to form a well-mixed sol-gel. The prepared sol-gel was initially dried at 100 °C for 24 h, followed by vacuum drying at the same temperature for another 24 h. Finally, the solid product was calcined in air at 700 °C for 1 h, with a heating rate of 5 °C per minute (Scheme 1).

### 2.2. Photocatalytic NPs Doped PU NFs

PU solution (MW = 110,000, medical grade) with a concentration of 10 wt% was prepared by stepwise dissolving the PU in a mixture of tetrahydrofuran (THF) and N,N-dimethylformamide (DMF). Initially, the PU pellets were dissolved in THF overnight. Subsequently, DMF was added to create a final sol-gel solution containing 10 wt% PU in a 1:1 weight ratio of THF to DMF. Two colloidal suspensions were then prepared by separately adding 0.2, 0.3, and 0.6 g of Cd/ZnO NPs into 10 g of the PU solution, followed by stirring for 1 h at room temperature. The colloidal mixtures were loaded into a plastic syringe with a capillary tip for electrospinning. During the electrospinning process, a copper pin connected to a positive electrode (anode) was inserted into the solution, while a rotating drum covered with a polyethylene sheet served as the collector connected to a negative electrode (cathode). The electrospinning was carried out at a voltage of 20 kV, with a working distance of 15 cm between the syringe tip and the collector (Scheme 1). The resulting nanofiber mats were dried under vacuum at 40 °C for 24 h. The samples comprising 0.2, 0.3, and 0.6 g of CdO-doped ZnO were designated as P1, P2, and P3, respectively.

### 2.3. Photohydrolysis H_2_ Production from AB

The experimental apparatus used to assess H_2_ photogeneration was set up according to the gas volumetric technique, as detailed in the literature [10]. Typically, a 50 mL Pyrex glass reactor was equipped with a water-jacketed cooling system. The reactor outlet was firmly attached to a gas burette for accurate evaluation of the photoproduced H_2_. A total of 100 mg of each CdO-doped ZnO@PU photocatalyst was introduced into the reactor, followed by the addition of a predetermined volume of a 1 mmol AB aqueous solution. The system was then irradiated with visible light (λ > 420 nm) to stimulate photocatalytic performance. A magnetic stirrer facilitated uniform mixing of the solution, while a water circulator accurately regulated the reaction temperature to maintain stable conditions. The volume of photogenerated H_2_ was evaluated by measuring the amount of water displaced within the sealed system during the light exposure period. The reaction continued until no further H_2_ photoproduction was observed. To conduct a comparative study, the photocatalytic activity of the CdO-doped ZnO@PU photocatalyst was assessed under dark conditions to determine the role of visible light in H_2_ photoproduction performance. The effects of catalyst dosage, AB concentration, temperature, and light intensity were systematically studied.

### 2.4. Photocatalyst Characterization

The surface morphology of the hybrid nanofibers was examined using a JEOL JSM-5900 scanning electron microscope (JEOL Ltd., Tokyo, Japan) and a Hitachi S-7400 field-emission scanning electron microscope (FESEM, Tokyo, Japan). The phase and crystallinity of the catalyst were analyzed with a Rigaku X-ray diffractometer (Rigaku Co., Tokyo, Japan), employing Cu Kα radiation (λ = 1.54056 Å) over a 2θ range of 10° to 80°. For further analysis, transmission electron microscopy (TEM) was conducted at 200 kV, and EDX (JEOL Ltd., Japan) was used. The surface composition was detected using X-ray photoelectron spectroscopy (XPS, AXIS-NOVA, Manchester, UK) under the following conditions: base pressure of 6.5 × 10^−9^ Torr, resolution (pass energy) of 20 eV, and a scan step of 0.05 eV/step. The absorption spectra of CdO-doped ZnO NPs were examined utilizing an HP 8453 UV–vis spectroscopy system (Waldbronn, Germany).

## 3. Results and Discussion

### 3.1. Morphological Characterization

Electrospinning is a versatile technique for producing NFs from a wide range of materials, including polymers, metals, bimetals, and metal oxides, with numerous potential applications. This method utilizes an electric field to stretch charged polymer solutions or melts into ultrafine fibers, which are subsequently collected on either a rotating drum or a stationary collector. These NFs exhibit unique characteristics, including a high surface area-to-volume ratio, porosity, and mechanical strength, making them ideal for applications such as filtration membranes, tissue engineering scaffolds, sensors, and drug delivery systems.

**Figure 1:**
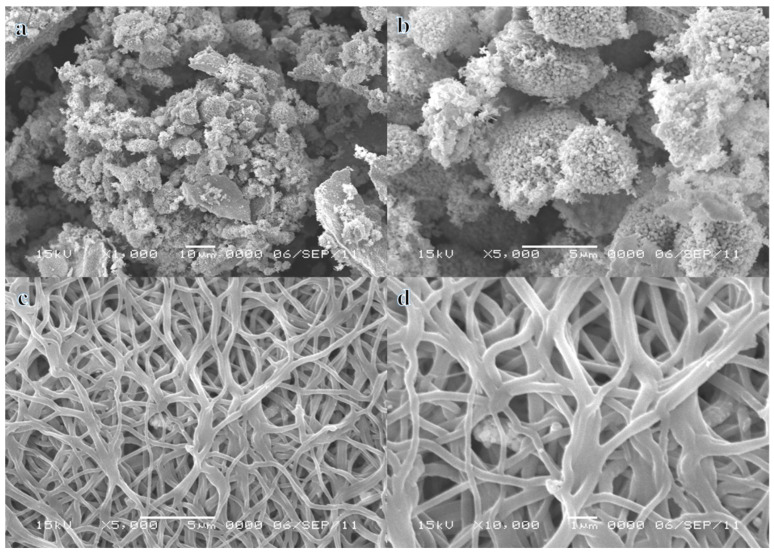
(**a**,**b**) SEM images of CdO-doped ZnO NPs after calcination at 700 °C for 1 h and (**c**,**d**) P3 NFs after drying at 40 °C for 24 h.

Recently, we expanded the scope of this technique by incorporating colloidal solutions into the electrospinning process. In this method, NPs are dispersed in the polymer solution before electrospinning, resulting in NFs with improved functionalities, such as enhanced electrical conductivity and catalytic performance. This method has shown promise in various fields, including flexible electronics, catalysis, and environmental remediation. Our study found that small-sized NPs were fully encapsulated within the polymer NFs. Figure 1a,b show SEM images of the prepared CdO-doped ZnO NPs after calcination, which appear as semi-ball-like NPs. Figure 1c,d illustrate SEM images of the P3 electrospun NFs, representing the maximum electrospinnable concentration. The fibers exhibited random orientation due to the bending instability of the spinning jet. This random structure contributes to a high surface area-to-volume ratio of the nanofibers, which is advantageous for applications that require efficient mass transfer.

Furthermore, the integration of NPs within the NFs enhances their overall functionality across various applications. The smooth and continuous surfaces of these randomly oriented fibers facilitate better interaction with surrounding materials, thereby improving their mass transfer capabilities. This combination of random orientation and embedded NPs enhances the versatility and effectiveness of these NFs in a wide range of applications. Notably, no visible NPs were observed on the surface of the PU nanofibers, indicating that the NPs were well-dispersed within the PU NFs. This uniform dispersion contributes to the consistent properties and performance of the NFs. Additionally, the absence of visible NPs suggests that the NPs are effectively encapsulated within the NF structure, promoting long-term stability and durability.

### 3.2. Phase Analysis

**Figure 2:**
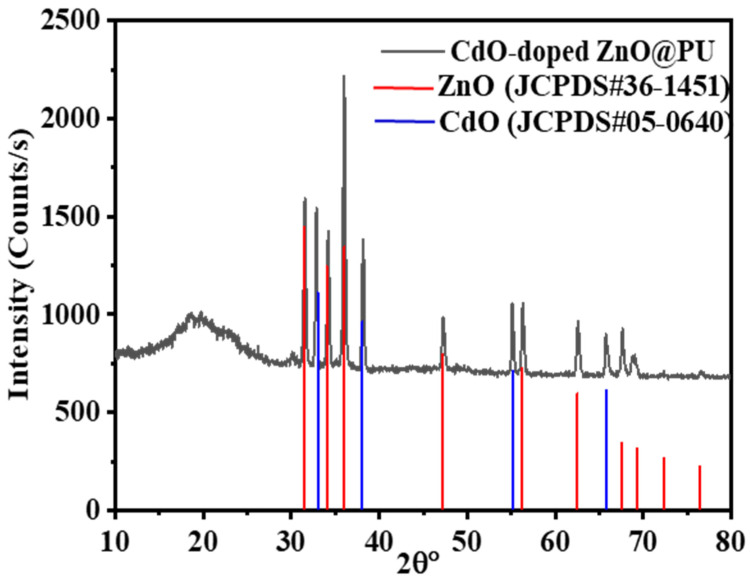
XRD patterns of the P3 nanocomposite [24].

Figure 2 presents the X-ray diffraction (XRD) patterns of the P3 composite. The intense diffraction peaks at 2θ values of 31.49°, 34.02°, 36.02°, 47.15°, 56.21°, 62.51°, 67.57°, 69.32°, 72.35°, and 76.40° correspond to the (100), (002), (101), (102), (110), (103), (112), (201), (004), and (202) planes, respectively, confirming the formation of a ZnO (zincite) phase with a hexagonal structure (space group P63mc (186), JCPDS#36-1451). This crystallographic information confirms the successful synthesis method employed for the NPs. The peaks observed at 2θ values of 33.00°, 38.04°, 55.20°, and 65.80° are associated with the (111), (200), (220), and (311) planes, indicating the formation of a cubic CdO phase (space group Fm3m (225), JCPDS#05-0640), which is attributed to CdO. Additionally, the broad peak around 19° is attributed to the PU matrix.

**Figure 3:**
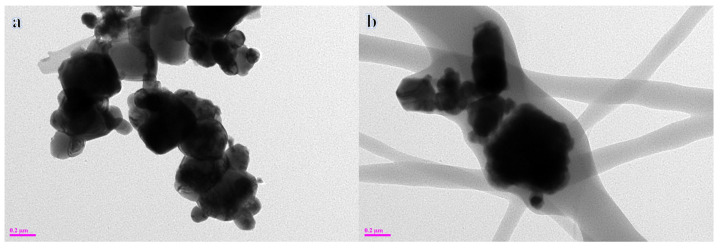
(**a**) TEM images of CdO-doped ZnO NPs after calcination at 700 °C for 1 h and (**b**) P3 NFs after drying at 40 °C for 24 h [24].

The morphological properties of the Cd/ZnO NPs and the P3 nanocomposite were investigated using TEM, as shown in Figure 3. The TEM images indicate that the Cd/ZnO nanocomposite comprises nanoscale, spherical-like particles (Figure 3a). The Cd/ZnO NPs are uniformly distributed and effectively incorporated within the nanofibers of the P3 composite (Figure 3b). This homogeneous embedding elucidates the lack of discernible NPs in the SEM pictures (Figure 1c,d) and substantiates the XRD results, which demonstrated the effective integration of the NPs into the polymer matrix. The structural integrity and uniformity evident in the TEM images underscore the efficacy of the fabrication technique in generating a robust and efficient photocatalyst.

**Figure 4:**
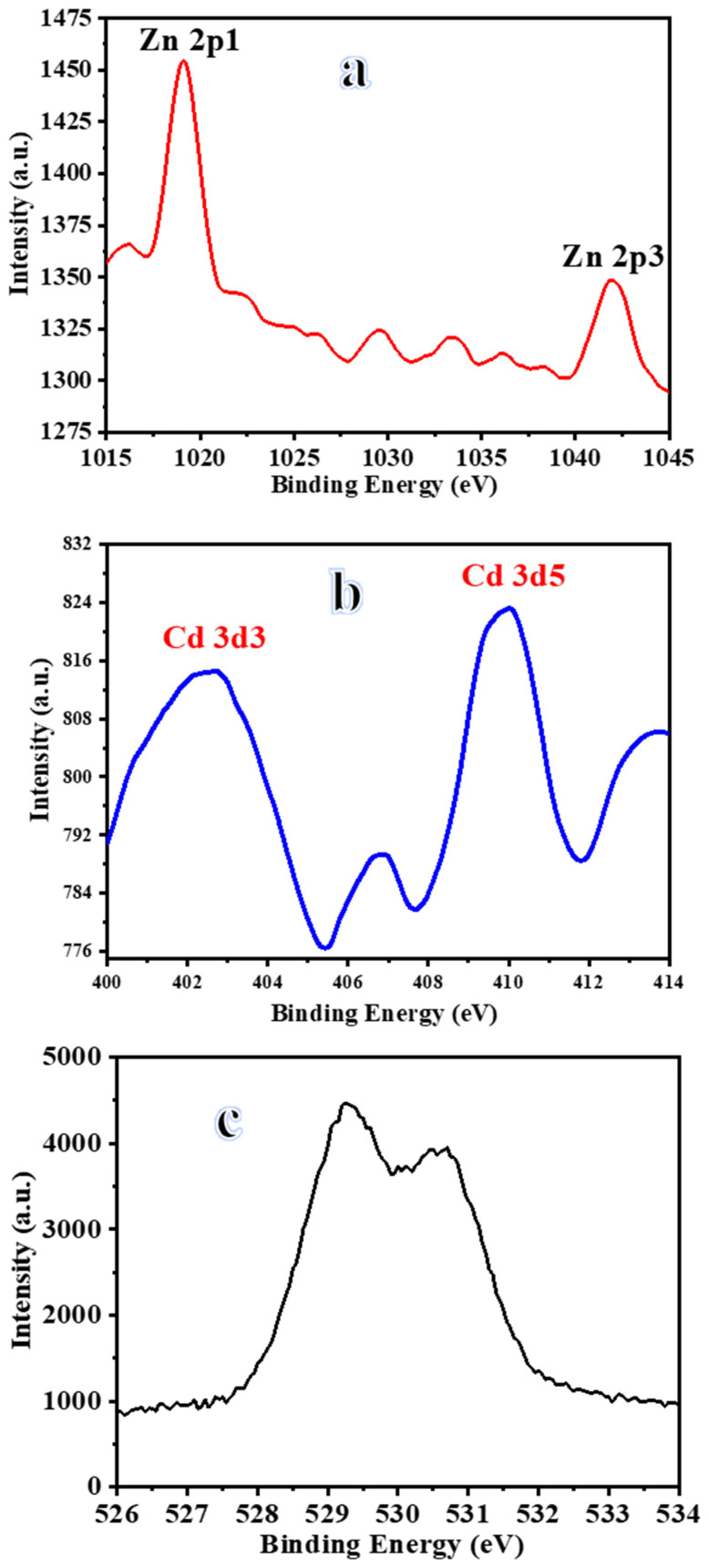
XPS spectrum of P3 nanocomposite (**a**) zinc, (**b**) cadmium, and (**c**) oxygen.

Figure 4 presents the XPS analysis of the P3 nanocomposite, revealing the chemical states of its constituent elements. In the Zn 2p spectrum (Figure 4a), two prominent peaks are observed at binding energies of 1019.13 eV and 1041.94 eV, corresponding to Zn 2p3/2 and Zn 2p1/2, respectively. These peaks confirm the presence of Zn^2+^ in ZnO, consistent with previous reports [25]. The Cd 3d spectrum (Figure 4b) displays peaks at 410.11 eV and 402.57 eV, attributed to Cd 3d3/2 and Cd 3d5/2, respectively, indicating the incorporation of Cd^2+^ in CdO [26,27]. In the O 1s spectrum (Figure 4c), two distinct peaks are observed at 529.31 eV and 530.60 eV. The peak at 529.31 eV is associated with lattice oxygen in CdO [28,29], while the peak at 530.60 eV corresponds to lattice oxygen in ZnO [30]. Additionally, the peak at 530.60 eV can also be attributed to O–H bonds, representing hydroxyl groups present on the surface of the nanocomposite [31]. The O 1s peak at 530.60 eV is further linked to O^2−^ ions in oxygen-deficient regions of the ZnO matrix, indicative of oxygen vacancies in the lattice structure. These oxygen vacancies are known to play a critical role in enhancing the catalytic activity of the prepared nanocomposite by facilitating improved charge carrier dynamics.

### 3.3. Optical Properties

**Figure 5:**
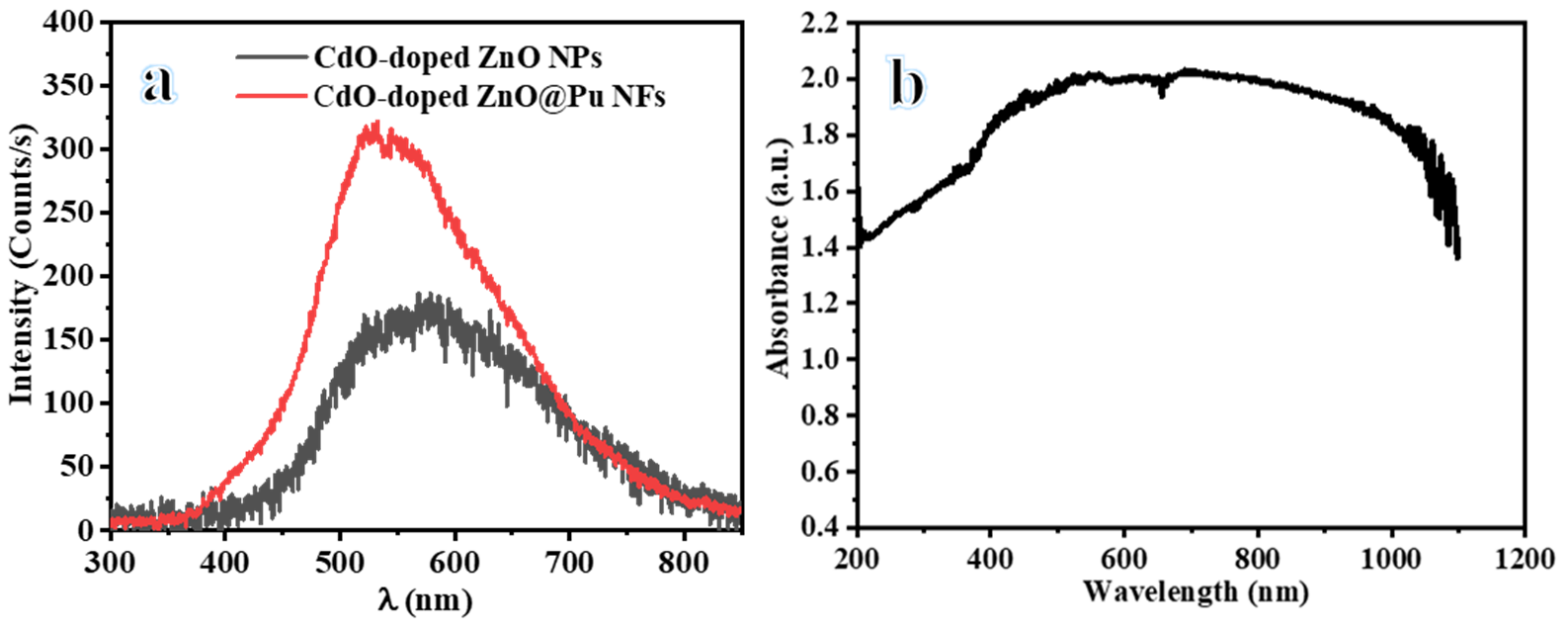
(**a**) PL spectra of the synthesized CdO-doped ZnO NPs and P3 nanocomposite [24] and (**b**) diffuse reflectance spectrum of CdO-doped ZnO NPs.

Photoluminescence (PL) emission is a widely used technique for investigating charge carrier behavior in semiconductors, offering valuable insights into charge trapping, migration, separation, and recombination. These factors significantly affect photocatalytic performance as they determine the efficiency of the material under light exposure. In this study, PL spectra were obtained for both CdO/ZnO NPs and CdO/ZnO-doped PU hybrid nanofibers at an excitation wavelength of 300 nm and at room temperature, as shown in Figure 5a. The observed emission peak around 530 nm in both samples reflects charge recombination dynamics, which can be used to measure the material’s photocatalytic performance. The emission at 530 nm suggests that charge carrier recombination occurs at defect sites within the material, likely due to oxygen vacancies or similar structural defects. These defects can act as trapping sites for electrons (e^−^) and holes (h^+^), thus extending their lifetimes and reducing the probability of recombination. This process improves the efficiency of photocatalytic reactions by allowing charge carriers more time to participate in surface redox reactions. The estimated band gap energy, calculated using the formula E_(eV)_ = hc/λ_g_, is approximately 2.33 eV, where λ_g_ represents the absorption edge of the photocatalyst. This relatively low band gap indicates that the material is responsive to visible light, making it suitable for photocatalytic applications under visible light irradiation. The obtained band gap is compared with several CdO-ZnO samples prepared using different methods, as reported in the literature (Table 1). Furthermore, the low band gap energy implies a reduced recombination rate of e^−^ and h^+^, which enhances the photocatalytic performance of the material. The high separation and transfer rates of e^−^ and h^+^, as indicated by the PL spectra, suggest that the prepared photocatalysts exhibit stable and efficient photocatalytic activity. This stability and efficiency can be attributed to the effective separation of e^−^ and h^+^ in the doped NPs, which decreases the possibility of recombination and increases the likelihood of catalytic reactions occurring on the photocatalyst surface. The ability to efficiently transfer charge carriers further supports the material’s potential for sustained photocatalytic activity.

Figure 5b displays the UV–vis absorption spectra of the CdO/ZnO NPs, indicating a wide absorption range from 400 to 1100 nm within the visible light spectrum. The improved level of absorption can be attributed to the synergistic interaction between CdO and ZnO, which promotes a reduction in the band gap and broadens the light-harvesting spectrum. The existence of oxygen vacancies within the lattice structure significantly enhances the optical characteristics of the CdO/ZnO NPs. These oxygen vacancies generate localized electronic states within the band gap, facilitating enhanced light absorption and augmented photocatalytic activity under visible light.

### 3.4. Photohydrolysis of AB

**Figure 6:**
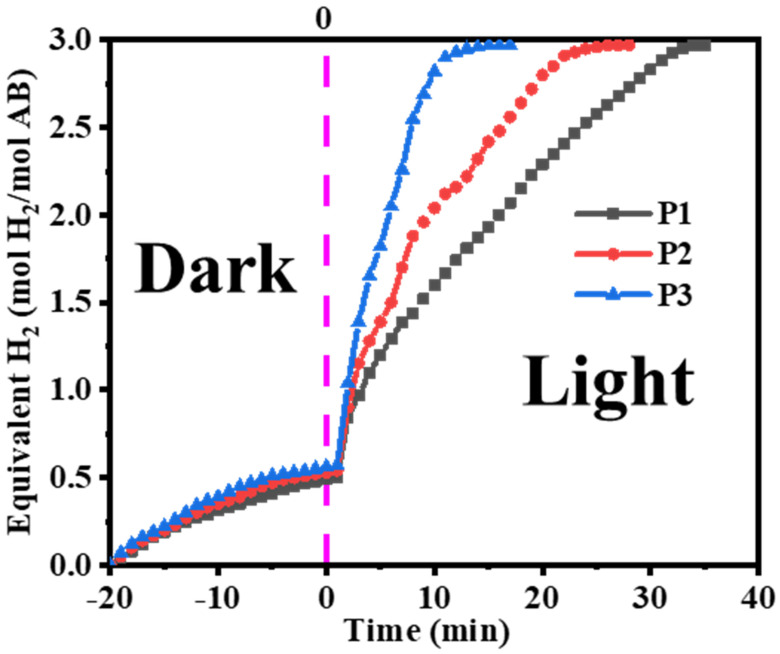
The effect of various photocatalytic nanocomposites on H_2_ photoproduction.

The evolution of H_2_ from AB under visible-light irradiation and dark conditions was studied through a series of experiments using CdO-doped ZnO NPs@PU NFs. At 25 °C, the photocatalytic activity of these nanocomposites was tested by observing the photoevolution of H_2_ from an aqueous solution containing 1 mmol of AB. This experiment was conducted simultaneously using 100 mg of various samples, designated as P1, P2, and P3. Figure 6 describes the photoproduction of H_2_ as a function of irradiation time for P1, P2, and P3. A significant increase in the photoevolution of H_2_ was observed upon activation of the light source, confirming the photocatalytic activity of the introduced nanocomposite photocatalysts. Over time, the H_2_ production reached stoichiometric H_2_ in AB (~67 mL), which is equal to 3 mmol of H_2_, coinciding with the full photodehydrogenation of AB. The rate of H_2_ evolution was shown to be directly affected by the loading of CdO-doped ZnO NPs embedded in PU NFs. A significant increase in H_2_ production was observed when the nanocomposites were loaded with a greater amount of photocatalysts. Specifically, the P3 NFs demonstrated the maximum photocatalytic activity, resulting in approximately 100% H_2_ yield within a 15 min period. In contrast, the P1 NFs yielded 64%, while the P2 NFs yielded 80% under identical conditions and timeframe. The P3 NFs exhibited a good TOF of 31.2 min^–1^, surpassing values reported in the literature (Table 2). These results provide compelling evidence of the synergistic effects resulting from the combination of CdO and ZnO NPs. Furthermore, the enhancement of H_2_ evolution can be attributed to improved charge carrier separation and the extension of band gap absorption into the visible light range. Additionally, as shown in Figure 6, a significant reduction in H_2_ generation was observed when the photohydrolysis process was catalyzed by the nanocomposites in the absence of light.

**Figure 7:**
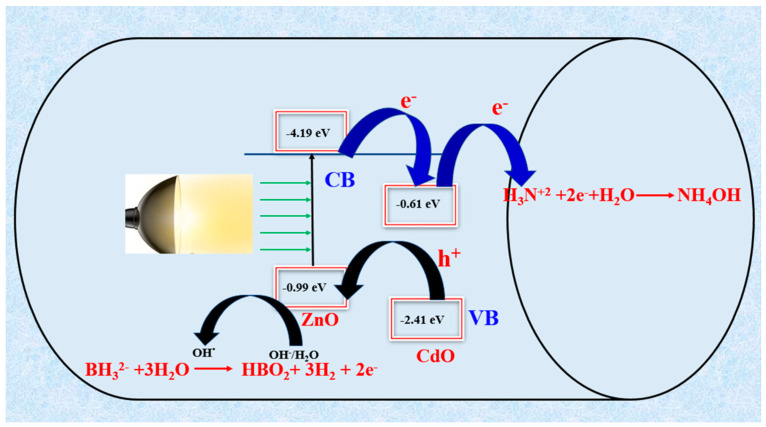
Schematic demonstrating the photodehydrogenation of AB using P3 NFs.

The increased catalytic activity observed during AB photodehydrogenation under visible light irradiation can be ascribed to the proposed reaction pathway, as illustrated in Figure 7. At 25 °C, AB manifests as a stable solid due to the variations in electronegativity among nitrogen, boron, and H_2_. In the AB molecule, the nitrogen atom, owing to its higher electronegativity, draws electrons from the adjacent H_2_ atoms, resulting in partial positive charges on the H_2_ atoms. Conversely, the boron atom, due to its electropositive nature, relinquishes electrons to its corresponding H_2_ atoms, leading to the formation of partial negative charges. This interaction results in the creation of dihydrogen bonds, which, while weaker than ionic or covalent connections, are essential for preserving the structural integrity of the AB molecule. The comparatively weak nature of dihydrogen bonds allows for their facile disruption in electron-rich environments, thereby promoting the onset of hydrolysis. The photohydrolysis of AB using CdO-doped ZnO NPs@PU NFs is posited to facilitate effective photodehydrogenation when exposed to visible light irradiation. The dissociation of photogenerated electron−hole (e^−^-h^+^) pairs, induced by visible light absorption, is crucial to this process. This mechanism is intricately associated with the conduction band (CB) and valence band (VB) potentials of the CdO and ZnO constituents in the nanocomposite, which regulate charge carrier dynamics and dictate the efficiency of electron transfer. The energies of the CB and VB of the component materials were estimated using empirical formulas, elucidating their significance in the overall reaction process [41]:

E_CB_ = χ − E_e_ − 0.5E_g_ (2)

E_VB_ = E_CB_ + E_g_ (3)

Equation (1) defines the relationship between CB energy (E_CB_), electron energy (E_e_), and the bandgap energy (E_g_) for E_CB_. Similarly, Equation (3) describes the connection between VB energy (E_VB_) and E_CB_, factoring in the E_g_. The energy of the electrons, referenced against the normal hydrogen electrode (NHE), is denoted by E_e_ and is measured at 4.5 eV. The E_g_ represents the energy difference between CB and VB of the semiconductor, while χ denotes the semiconductor’s electronegativity. Based on previous findings, the conduction and VB potentials of CdO relative to the SHE were calculated to be −4.61 eV and −2.41 eV, respectively. CdO’s bandgap, at 2.20 eV, was significantly narrower than that of ZnO, which had a bandgap of 3.20 eV. Consequently, CdO’s VB lies at a higher energy level (−2.41 eV) compared to that of ZnO (−0.99 eV), facilitating efficient charge transfer from the VB of CdO to ZnO. Upon photoexcitation, the photogenerated holes (h^+^) react with water or hydroxide ions (OH^−^), resulting in the generation of hydroxyl radicals (OH^•^). The positive charges accumulated on the VB of the ZnO surface attract the H_3_B^2−^ anions from AB, initiating an oxidation reaction as described in the following mechanism. The band alignment between CdO and ZnO plays a crucial role in promoting the separation and transfer of charge carriers, thereby enhancing the overall photocatalytic activity under visible light irradiation.

H_3_B^2−^ + 3H_2_O → HBO_2_ + 3H_2_ + 2e (4)

It is important to note that the CB of CdO, positioned at −4.61 eV, lies at a lower energy level than that of ZnO, which is at −4.19 eV. Upon exposure to visible light, electrons from the VB of ZnO are excited and transition to ZnO’s CB, creating vacancies (holes) in the VB. These excited electrons in the CB of ZnO are subsequently transferred to the lower energy CB of CdO, facilitating efficient charge separation. This charge transfer highlights the potential of CdO as an effective sensitizer capable of utilizing visible light for photocatalysis. The high mobility of the photogenerated electrons in CdO ensures their active participation in the photohydrolysis of AB, demonstrating a high degree of electron−hole separation with minimal recombination. The surface of CdO becomes electron-rich, enhancing its ability to drive hydrolytic photohydrolysis reactions. During the photocatalytic process, the photogenerated electrons are transferred from the CB of ZnO to the CB of CdO, resulting in electron accumulation in CdO and the creation of holes (positive charge carriers) in ZnO. As AB adsorbs onto the surface of the PU matrix, the nitrogen atom in the N–B bond of AB serves as an electron donor. The accumulated electrons in CdO can then be transferred to the positively charged H_3_N^2+^ species in AB, facilitating the subsequent oxidation reaction. This electron transfer mechanism underscores the role of CdO-doped ZnO nanocomposites as efficient photocatalysts for H_2_ generation through photodehydrogenation.

H_3_N^2+^ + 2e + H_2_O → NH_4_OH (5)

It is important to highlight that the overall reaction mechanism closely aligns with the established photohydrolysis process of AB, as illustrated in the following equation.

NH_3_BH_3_ + 3H_2_O → NH^+4^ + BO^−2^ + 3H_2_ (6)

**Figure 8:**
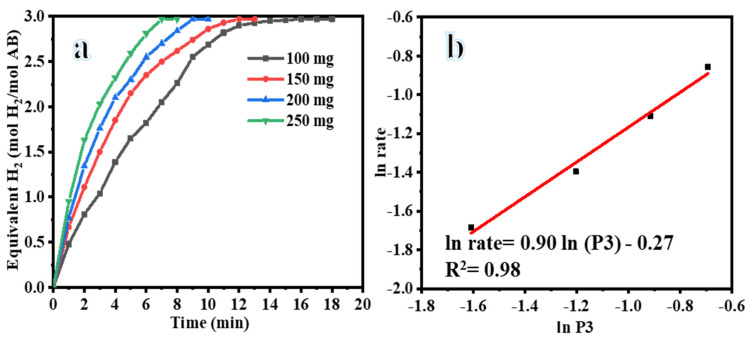
The effect of P3 dosages on H_2_ photoproduction (**a**) and logarithmic correlation between the rate of H_2_ photoevolution and P3 (**b**).

The P3 nanocomposite, which exhibited the best catalytic performance in this study, was chosen for further examination of the kinetics of AB photodehydrogenation by varying the P3 dosage, AB concentration, and reaction temperature. We examined the impact of the P3 nanocomposite dose on the H_2_ photoproduction rate using 1 mmol of AB, with dosages ranging from 100 mg to 250 mg, under 25 W/m^2^ light irradiation at 25 °C (Figure 8a). The results demonstrated a rapid and nearly linear photogeneration of H_2_, yielding a total volume of approximately 67 mL, likely attributable to the enhanced surface area of P3 that facilitated the AB photodehydrogenation process. Increasing the P3 dosage led to a reduction in the duration of H_2_ photorelease, achieving 100% yield in 16, 12, 9, and 7 min for 100, 150, 200, and 250 mg of P3, respectively. A logarithmic plot of the H_2_ photoproduction rate versus the P3 dose further elucidated the reaction kinetics (Figure 8b). The reaction rate increased with higher P3 doses, suggesting that the enhancement in AB photodehydrogenation may be attributed to the increased P3 dosage. This enhancement could be due to the rise in the number of accessible catalytic sites on the photocatalytic nanocomposite surfaces. A slope of 1.03 in Figure 8b indicates a first-order reaction concerning P3 loading in the photodehydrogenation process. These findings demonstrate that the H_2_ photogeneration rate escalated linearly with increasing dosages of the P3 nanocomposite, suggesting the significance of catalyst loading in facilitating AB photodehydrogenation under visible light.

**Figure 9:**
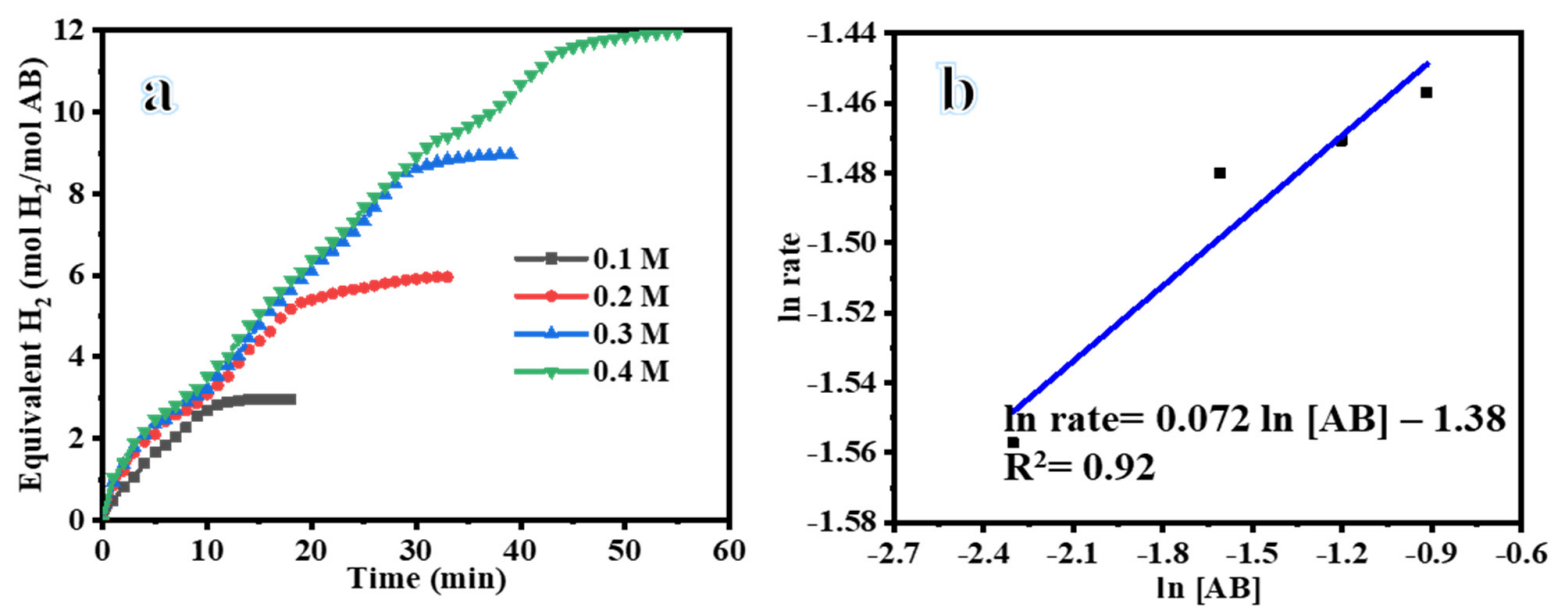
Effect of [AB] on the photoevolution of H_2_ (**a**) and H_2_ photogeneration rate versus [AB] (**b**).

Figure 9a shows the H_2_ photoevolution profile from AB photodehydrogenation using 100 mg of the P3 nanocomposite at different concentrations of [AB] under 25 W/m^2^ illumination and at a temperature of 25 °C. The rate of H_2_ production remains constant and is mostly unaffected by the concentration of [AB]. Notably, the concentration of [AB] did not influence H_2_ production; the initial H_2_ production mostly remained constant despite the increase in [AB]. Figure 9b presents a plot of H_2_ photoevolution versus [AB] on a logarithmic scale. The slope of this plot was 0.04, indicating that the reaction is zero-order with respect to the concentration of AB. These data suggest that the photocatalyst’s activity plays a more crucial role in enhancing H_2_ photogeneration rates than the concentration of [AB].

**Figure 10:**
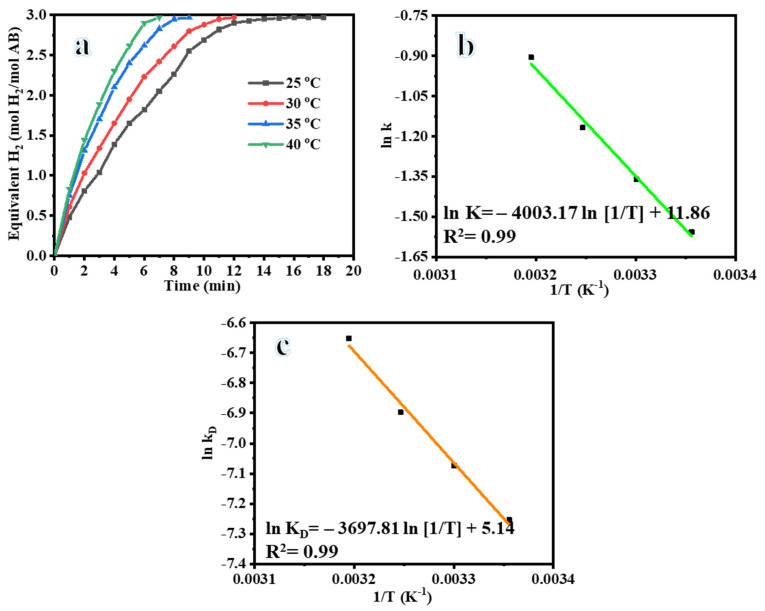
The influence of temperature on the photoevolution of H_2_ (**a**), correlation of logarithmic K and 1/T (**b**), and correlation of logarithmic K_D_ and 1/T (**c**).

The influence of temperature on AB photodehydrogenation, augmented by P3 nanocomposites, was investigated over a temperature range of 25 °C to 40 °C (Figure 10a). The study utilized 1 mmol of AB and 100 mg of P3 under 25 W/m^2^ light intensity. The results demonstrate a significant enhancement in H_2_ generation with rising temperature due to improved charge carrier mobility and interfacial charge transfer. As the reaction temperature increases, the mobility of photoelectron−hole pairs intensifies; electrons can interact more rapidly with adsorbed oxygen, while holes can more swiftly form OH radicals alongside −OH groups, thereby enhancing AB photodehydrogenation [54,55,56]. The reaction rate increases with elevated temperatures, demonstrating that the efficiency of AB photodehydrogenation improves due to enhanced charge mobility at higher temperatures. The rate constant k was derived from the slope of the linear segment of the plots, and an Arrhenius plot (Figure 10b) was used to calculate the Ea of the reaction, which was found to be 32.28 kJ/mol. This minimal Ea indicates the efficient catalytic efficacy of the P3 nanocomposites. Additionally, the Eyring plot (Figure 10c) was employed to determine the activation enthalpy ΔH and activation entropy ΔS for the synthesized photocatalyst, yielding values of 32.82 kJ mol^−1^ and 53.03 J mol^−1^ K^−1^, respectively. These results demonstrate that P3 nanocomposites exhibit significant catalytic performance for H_2_ photoproduction from AB.

## 4. Effect of Light Intensity

**Figure 11:**
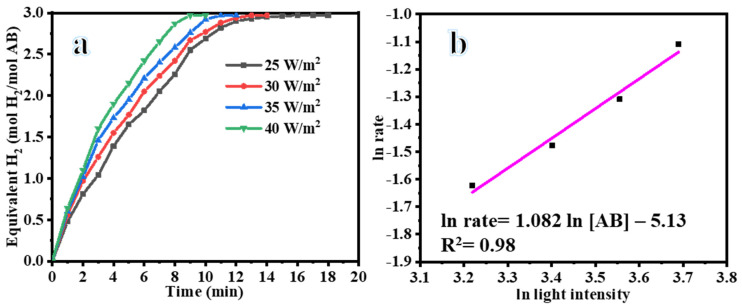
The influence of light intensity on the photoevolution of H_2_ (**a**) and H_2_ photogeneration rate versus light intensity (**b**).

The photogeneration of H_2_ during the photodehydrogenation of AB is influenced by both light intensity and wavelength [57,58]. Figure 11a illustrates the effect of light intensity on the AB photohydrolysis using 100 mg of the P3 nanocomposite and 1 mmol of AB at 25 °C. The collected data indicate that the rate of AB photodehydrogenation increases with higher light intensity. An increase in light intensity likely corresponds to a higher photon number, thereby enhancing the AB photohydrolysis. The rate constant increases with higher light intensities, showing an intensification of the photodehydrogenation of AB as the light intensity rises (Figure 11b). The plot’s slope was 1.082, indicating that the reaction is first order with respect to light intensity.

## 5. Reusability Test

**Figure 12:**
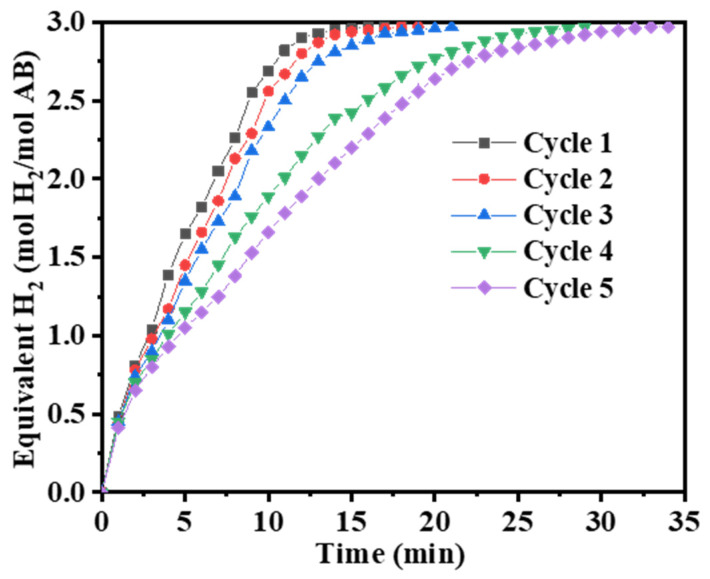
Reusability of prepared P3 NFs.

Figure 12 illustrates the recyclability assessment of P3 nanofibers, indicating that these nanofibers were used in the photogeneration process on five separate occasions. The photoproduced H_2_ exhibited negligible reduction during the first three cycles, while a slight drop was observed thereafter. This decrease may be attributed to the increased viscosity of the reaction solution or the deactivation effect resulting from increased metaborate content [46]. In comparison to other nanostructures, nanofibers have a reduced surface area, which may result in diminished performance as catalyst support. Nonetheless, it has been shown that the elongated axial ratio of the nanofibrous shape provides a significant benefit, markedly improving performance relative to other structures.

## 6. Conclusions

This study illustrates the effectiveness of CdO/ZnO NPs@PU NFs as a powerful and efficient photocatalyst for H_2_ photogeneration from AB under visible light irradiation. The P3 composite, which contains the highest concentration of CdO/ZnO NPs, demonstrated outstanding photocatalytic performance, achieving 100% H_2_ photogenerated within 15 min. This good photocatalytic activity can be attributed to the efficient separation of photogenerated charge carriers and the increased absorption of visible light facilitated by the unique characteristics of CdO and ZnO.

Kinetic studies demonstrated a first-order dependency on catalyst dosing and a zero-order dependency on AB concentration, with an Ea of 32.28 kJ/mol. These findings offer a sustainable and efficient method for photocatalytic H_2_ generation.

## Figures and Tables

**Table 1:** Comparison of band gap of different CdO-ZnO samples prepared using various methods.

Sample	Fabrication Method	Bandgap (eV)	Reference
1	Sol-gel method	3.20	[32]
2	Pulsed laser deposition	2.4	[33]
3	Sol-gel spin coating	2.66	[34]
4	Reflux method	3.18	[35]
5	Sol-gel spin coating	2.4	[36]
6	High-frequency reactive magnetron	2.82	[37]
7	Pulsed laser deposition	2.97	[38]
8	Spray pyrolysis method	2.82	[39]
9	Thermal decomposition	2.75	[19]
10	Vapor-to-solid mechanism	2.99	[40]
11	Co-precipitation technique	2.97	[41]
12	Electrospinning	2.62	[42]
13	Sacrificial template accelerated hydrolysis	2.81	[43]
14	Wet chemical method	3.2	[44]
15	Electrospinning	2.33	This study

**Table 2:** Catalytic performance of various catalysts in H_2_ photogeneration from AB.

Catalyst	TOF (min^–1^)	Reference
Co(0)@graphene	13.80	[45]
NiMo	27.30	[46]
CuCo@carbon	45	[47]
Bimetallic CoNi@graphene	16.40	[48]
Cu@Co@reduced graphene	8.36	[49]
CuCo NPs@functionalized graphene	15.46	[50]
NiCo@graphene oxide	6.78	[51]
Ni NPs supported on carbon nanotube	26.20	[52]
Ni NPs-carbon nanotube hybrid	23.50	[53]
CdO-doped ZnO@PU	31.2	This study

## Data Availability

The original contributions presented in the study are included in the article, further inquiries can be directed to the corresponding author.
